# A novel 1-channel EEG marker may associate with perioperative cognitive change

**DOI:** 10.3389/fnsys.2026.1688834

**Published:** 2026-05-26

**Authors:** Dana Baron Shahaf, Gil Bolotin, Oved Cohen, Mahli Raad, Raphael Derman, Goded Shahaf

**Affiliations:** 1 Department of Anesthesia, Rambam Health Care Campus, Haifa, Israel; 2 Bruce and Ruth Faculty of Medicine, Technion, Israel Institute of Technology, Haifa, Israel; 3 Department of Cardiac Surgery, Rambam Health Care Campus, Haifa, Israel; 4 Applied Neurophysiology Lab, Rambam Health Care Campus, Haifa, Israel

**Keywords:** attention markers, cardiac surgery, EEG, Montreal cognitive assessment (MoCA), postoperative cognitive dysfunction

## Abstract

**Introduction:**

Postoperative cognitive decline, at least transiently, is considered a prevalent and significant complication in elderly patients undergoing surgery. If the mechanisms that underlie it impact brain function intraoperatively, an effective intraoperative marker of brain dysfunction would be useful. However, to date, there is no consensus marker for this aim. We have developed an EEG-based marker—the Brain Reactivity Index (BRI). To assess the efficacy of intraoperative BRI as a marker of acute brain dysfunction that may relate to perioperative cognitive change, we compared its values between patients who showed different ranges of perioperative change in Montreal Cognitive Assessment (MoCA) scores.

**Methods:**

This study analyzed EEG and clinical data from 100 adult cardiac surgery patients to assess whether there might be an association between changes in the Brain Reactivity Index (BRI) and postoperative cognitive changes.

**Results:**

The intraoperative BRI was associated with perioperative MoCA changes in older patients (≥65 years), particularly in those with an intermediate (between 21 and 25) preoperative MoCA score. This association was apparent within the first 30 min of anesthesia. In contrast, no such association in BRI values was observed in the younger patient group (<65 years). The proportion of patients with cognitive dysfunction was comparable between the older and younger groups.

**Conclusion:**

These findings suggest that brain dysfunction, which is associated with perioperative cognitive decline, in older patients may often occur immediately after the induction of anesthesia, highlighting the potential for intraoperative monitoring and intervention. The comparable proportion of patients presenting with perioperative cognitive decline across age groups, along with the transient nature of this decrease in younger patients, suggests that the dysfunction would be temporary, whereas a separate factor may underlie persistent cognitive decline in older individuals.

**Clinical trial registration:**

NCT04512989

## Introduction

Delayed neurocognitive recovery is a common and significant complication affecting 30–50% of elderly patients undergoing surgery (Gold and Forryan, 2019; Evered and Silbert, 2018). Various theories have been suggested, and research continues to clarify the mechanisms underlying this often transient cognitive change (Brodier and Cibelli, 2021). A thorough understanding of the underlying causes is crucial for preventing delayed neurocognitive recovery and its potential progression into a long-term neurocognitive disorder in some patients.

Several mechanisms for perioperative cognitive decline have been suggested, with different onset times during the intraoperative and postoperative periods. For example, one proposed mechanism is an embolic shower (Hitchner et al., 2016), potentially resulting in either overt or covert stroke. Another mechanism involves hypoxia in specific brain vascular territories due to systemic hypoperfusion (Nakao et al., 2019). In both cases, a sudden intraoperative change in brain function may be expected to coincide with a specific surgical or anesthetic event. Conversely, a rapid adverse effect of anesthesia could lead to an abrupt change in brain function as early as the induction phase (Chuan et al., 2019), whereas a slower, potentially inflammatory effect of anesthesia or surgery might impact brain function only during the postoperative period (Danielson et al., 2020).

An effective intraoperative marker of brain dysfunction would be valuable if the mechanisms underlying perioperative cognitive change impact brain function already during surgery. Such a marker could enable the evaluation of potential intraoperative interventions aimed at reducing the incidence or severity of delayed neurocognitive recovery. Various modalities have been suggested for monitoring brain dysfunction (Yu et al., 2018), and EEG is one of the leading candidates (Punjasawadwong et al., 2018). However, to date, there is no consensus marker for this purpose (Chung et al., 2022). Brain dysfunction often involves EEG slowing (Kaszniak et al., 1979); however, similar slowing may be induced by anesthesia (Sloan, 1998) and may therefore not be sufficiently discriminative.

To enhance the differentiation between acute brain dysfunction and the baseline effects of anesthesia on the EEG signal, identifying EEG activity that is less influenced by anesthesia but remains sensitive to brain dysfunction is essential. We have demonstrated that effective anesthesia can significantly impact sustained attention (Baron Shahaf et al., 2022), while suggesting that its influence on earlier and shorter cognitive processes, such as perception or selective attention, may be considerably less pronounced (Baron Shahaf et al., 2020). This finding aligns with the distinction between sustained attention and selective attention in Posner’s model of attention (Posner and Boies, 1971). Excessively deep anesthesia may also reduce selective attention-related activity (Baron Shahaf et al., 2020), and such a reduction may also follow an acute brain dysfunction under anesthesia (Baron Shahaf and Shahaf, 2024a). Both the lasting activity, which underlies sustained attention, and the shorter activity, which may underlie selective attention, are spread over multiple brain regions (Shahaf et al., 2015), making them potentially measurable in the prefrontal region.

Over the last decade, we have been developing real-time markers for sustained attention, based on variability in a single prefrontal channel activity over a timescale of seconds. These markers have been validated by us and by other groups using thousands of samples from multiple clinical populations [a rather partial list: (Shahaf et al., 2017; Isserles et al., 2018; Shahaf et al., 2018; Gvion et al., 2021; Keren et al., 2024)], also under anesthesia (Baron Shahaf et al., 2022). To measure the shorter activity dynamics, rather than the lasting sustained attention-related activity (Shahaf et al., 2015), we monitored instead, in the current study, the variability at the resolution of up to a few hundred milliseconds, using the novel Brain Reactivity Index (BRI). Following the rationale presented above, we hypothesized that acute brain dysfunction under anesthesia may impact this short variability.

To evaluate the efficacy of the intraoperative BRI as a marker of acute brain dysfunction, we analyzed a cohort of patients who underwent cardiac surgery to evaluate a possible association between BRI values and the perioperative change in Montreal Cognitive Assessment (MoCA) scores. Comparisons were conducted separately for older patients (≥65 years) and younger patients (<65 years), given that delayed neurocognitive recovery is recognized as an age-related dysfunction (Sauër et al., 2009).

The concept of perioperative neurocognitive dysfunction, and even the more humble delayed neurocognitive recovery, is not sufficiently well-defined operationally (Rudolph et al., 2010). Therefore, as a first step of screening of the BRI, we compared it in this study only to perioperative MoCA changes. The precise characterization of the association between perioperative changes in the assessment score and brain dysfunction is not trivial, because there is a known effect of improvement in the assessment due to its repetition within a short time window (Duff et al., 2012; Baron Shahaf and Shahaf, 2024b). We therefore implemented an analysis that does not presume *a priori* a known threshold of the expected perioperative cognitive change that relates to brain dysfunction.

## Methods

### Patients

The study was approved by the Rambam Health Care Campus Institutional Review Board (IRB #542–20), and written informed consent was obtained from all subjects participating in the trial. The trial was registered before patient enrollment at clinicaltrials.gov (NCT04512989, Principal investigator: Dr. Dana Baron Shahaf, Date of registration: 14 August 2020). A total of100 patients who underwent elective cardiac surgery at Rambam Healthcare Center, between April 2021 and December 2022, were included in this study. All patients of any age who underwent elective cardiac surgery at Rambam during the study period and who agreed to sign an informed consent were eligible to participate. Exclusion criteria included a lack of informed consent, the inability to perform the MoCA test (also due to an overt confusional state, e.g., in postoperative delirium), major psychiatric disability, and major auditory or visual disabilities. The patients were divided *a priori* into two age groups: older (≥65 years) and younger (<65 years). We obtained the necessary preoperative and postoperative clinical data, as well as intraoperative EEG samples from 58 older patients and from 35 younger patients.

### Procedure

The patients underwent cognitive assessment twice, using the Montreal Cognitive Assessment (MoCA) (Nasreddine et al., 2005), once 1 day before surgery and again during the first postoperative week, 1 day before discharge from hospitalization. The assessments were administered by three trained evaluators. For each patient, both the preoperative and postoperative tests were administered by the same evaluator. Each test report was also reviewed by a trained physician. A majority of the patients who showed a perioperative MoCA decrease of 2 or more points underwent a third MoCA evaluation approximately 6 weeks after surgery. The same MoCA forms were used in each evaluation (preoperative, postoperative, and when relevant, ~6 weeks after surgery). In conjunction with each cognitive assessment, the levels of the patient’s anxiety and depression were evaluated using the Hospital Anxiety and Depression Scale questionnaire (HADS) (Zigmond and Snaith, 1983).

Approximately 80% of the patients in the study underwent coronary artery bypass graft surgeries (CABGs), and the remaining patients underwent valve replacement surgeries or combined CABG and valve replacement surgeries. The anesthesia protocol generally involved induction with etomidate, rocuronium, and fentanyl, and maintenance with 1 MAC Sevoflurane. EEG was recorded throughout the surgery, starting approximately 5 min before induction, using the Medtronic BIS monitor. After the surgery, the raw EEG data were exported from the BIS monitor to a flash drive for offline analysis. The BIS index was not sampled.

### BRI computation

**Figure 1:**
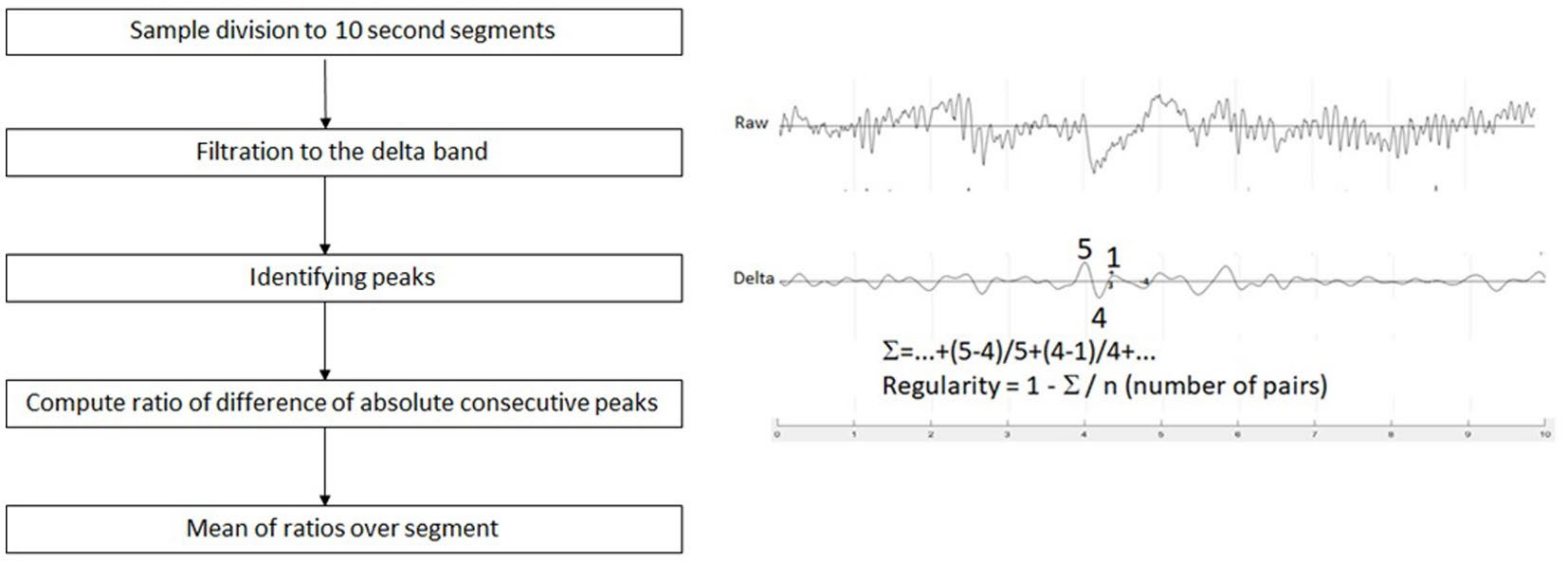
A schema of the BRI calculation. Peaks are local minima (smaller than their preceding and succeeding values) and local maxima (larger than their preceding and succeeding values).

Similar to our sustained attention marker (Baron Shahaf et al., 2022), each 10-s segment was filtered to the delta band (using a Python implementation of EEGLAB’s EEGFILT FIR filter). Then, the wave peaks (both positive and negative) were identified in the filtered segment. While the CEI sustained attention marker assesses variability over longer periods, the BRI assesses the variability on the hundred-millisecond scale. Thus, for every two consecutive peaks, we computed their absolute values, and the difference between these absolute values was divided by the largest of the two peaks. Then, the BRI marker for the segment was derived from one minus the average of these consecutive peak ratios and lay in the [0,1] range. See Figure 1 for a schematic description of BRI calculation. Segments in which the standard deviation of all the absolute peaks was greater than the mean were rejected as noisy, and no BRI value was returned. For each sample, we computed the average BRI. We excluded from further analysis samples that did not contain at least 5 min of valid BRI values (or 30 values, as BRI values are computed every 10 s). Less than 5% of the sample data were rejected as noisy.

### Statistical analysis

Based on previous computations, the BRI does not generally demonstrate a normal distribution. Therefore, the Kruskal–Wallis test (with a Bonferroni correction for multiple comparisons) or the Mann–Whitney U test was used (depending on the number of compared groups) for the assessment of significant differences in BRI between patients with various ranges of perioperative MoCA change. For the assessment of differences in various demographic and clinical variables with known normal distribution, the *t*-test was used. For comparison of ratios, we used the chi-square test.

## Results

**Figure 2:**
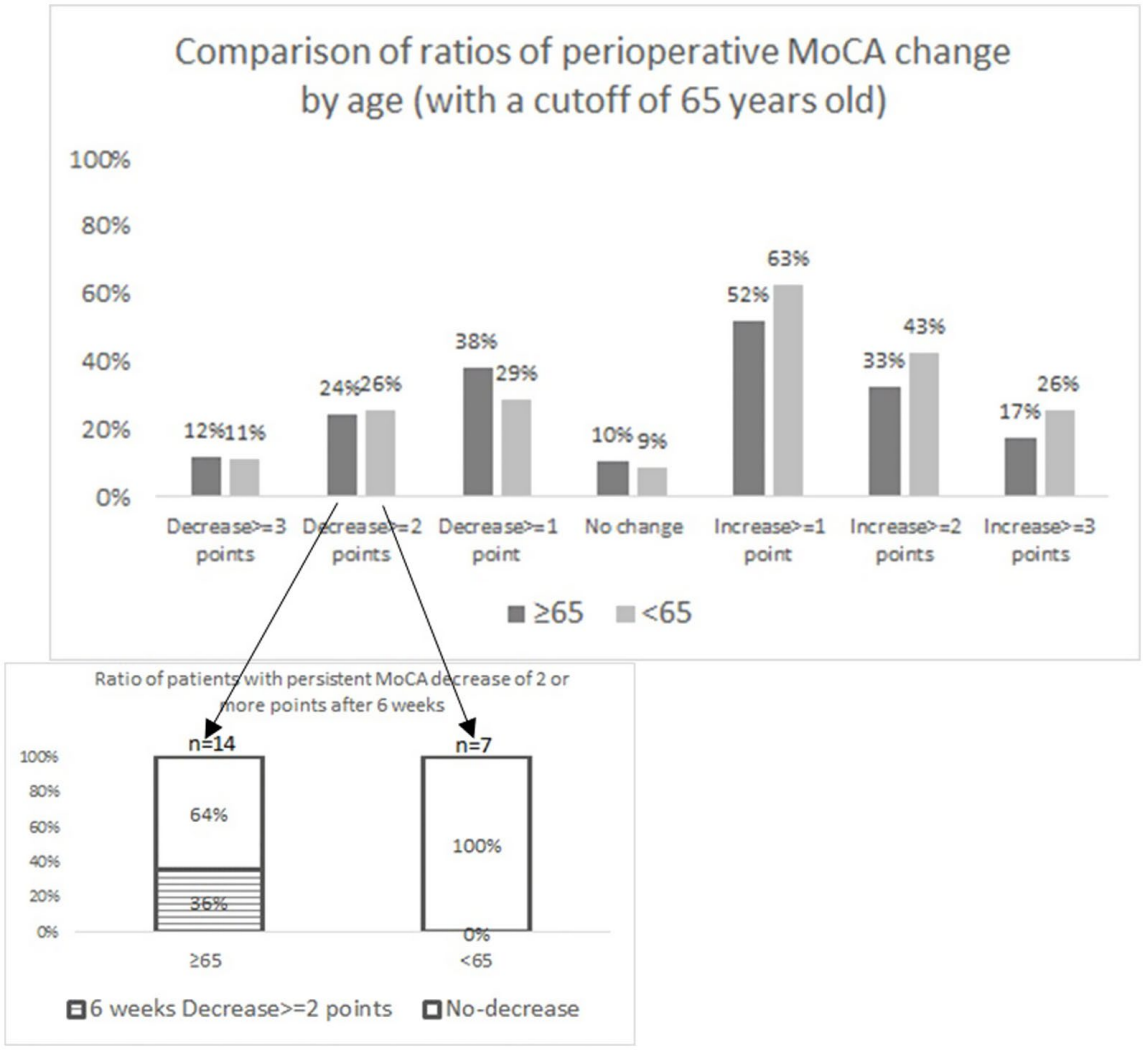
Ratio of patients with different thresholds of perioperative MoCA score change. Main figure: Thresholds range from 3 or more MoCA points perioperative change, of either increase or decrease, to 1 (any) MoCA point change. The ratios are presented for the older and younger groups. The ratio of patients without perioperative MoCA change is presented in the middle column. Inset: Ratios of persistent perioperative MoCA decrease after 6 weeks in the patients who showed at least 2 points of perioperative MoCA decrease (and who were also sampled after 6 weeks).

Figure 2 shows, in the main graph (upper), the percentages of patients who showed a decrease or increase in MoCA score between the preoperative and postoperative assessments. The percentages are presented for the two age groups cumulatively, with thresholds of either 1, 2, or 3 points of perioperative change. All patients with at least a 3-point change are also included in the group of patients with at least a 2-point change, and all of these patients are also included in the group of patients with at least a 1-point change. Notably, only 9–10% of the patients per age group, or nine patients altogether, did not demonstrate a perioperative MoCA change. As the definition of delayed neurocognitive recovery in terms of change in cognitive score is under debate and review (Rudolph et al., 2010), and to avoid undertaking assumptions in this regard in the current feasibility study, we focused the initial analysis with the patients who showed either a decrease or increase in perioperative MoCA score and included these nine patients with no perioperative change in their MoCA score in a secondary analysis. For the 84 patients in the initial analysis, we compared patients with a MoCA decrease of at least 1 point, of at least 2 points, and of at least 3 points and those with a MoCA increase of the same thresholds, respectively. Beyond the 3-point threshold in the older group and the 2-point threshold in the younger group, the sample sizes were too small (fewer than 5) to support meaningful comparison. Interestingly, the ratios of a 2-point decrease and of a 3-point decrease were rather similar for both age groups. The inset suggests a greater tendency toward a lasting decrease after 6 weeks in the older group; however, the sample sizes were too small for significant comparison. It should be noted that, in the case of no perioperative-related dysfunction, the MoCA score might be expected to improve with test–retest (Duff et al., 2012), and we demonstrated that this improvement is primarily in memory-related tasks, which the participants seem to learn through repeated exposure (Baron Shahaf and Shahaf, 2024b).

Table 1 presents the demographic and clinical preoperative, intraoperative, and postoperative characteristics of the patients, divided into groups by age and perioperative MoCA change. Within each age group, the data for patients who showed a perioperative MoCA decrease of at least 1 point are compared with those for patients who showed a perioperative MoCA increase of at least 1 point. There were no major differences except for the postoperative MoCA score (there was a significant difference in the younger group in the use of transfusion, but the number of patients involved was small). It should be noted that we did not rigorously evaluate postoperative delirium, but we excluded from the study two patients who were in an acute postoperative confusional state that prevented a reliable MoCA assessment.

**Table 1:** Demographic and clinical comparison of patients with MoCA decrease vs. increase.

Preoperative							
Age range (years)	Decrease or increase	Age (years) mean ± s.d.	Male: Female	Education (years) mean ± s.d.	Euroscore II	MoCA total mean ± s.d.	HADS total mean ± s.d.
≥65 [22 pts]	Decrease	71 ± 4	17:5	13.2 ± 3.6	4.1 ± 3.7	22.6 ± 4.0	9.8 ± 7.7
≥65 [30 pts]	Increase	71 ± 4	20:10	13.5 ± 3.4	4.2 ± 2.9	21.4 ± 2.8	10.0 ± 6.2
<65 [10 pts]	Decrease	54 ± 7	9:1	13.2 ± 3.8	3.2 ± 1.8	22.9 ± 2.8	10.9 ± 8.1
<65 [22 pts]	Increase	56 ± 9	19:3	13.4 ± 3.4	2.7 ± 2.0	22.5 ± 3.2	12.2 ± 6.3

Intraoperative				
Age range (years)	Decrease or increase	Surgery type (CABG: valves: combined)	CPB time (min) mean ± s.d.	Transfusion
≥65	Decrease	17:5:0	112 ± 45	4(/22)
≥65	Increase	23:6:1	108 ± 49	8(/30)
<65	Decrease	8:2:0	123 ± 44	3(/10)*
<65	Increase	17:4:1	127 ± 72	1(/22)

Postoperative				
Age range (years)	Decrease or increase	Postoperatively, the most prevalent complications 1. Atrial fibrillation 2. Need for packed cells	MoCA total mean ± s.d.	HADS total mean ± s.d.
≥65	Decrease	4 AF (/22)	19.8 ± 5.3**	11.6 ± 8.8
≥65	Increase	7 AF and 2 PC (/30)	23.6 ± 2.9	9.8 ± 7.7
<65	Decrease	1 AF and 1 PC (/10)	20.1 ± 3.6**	12.3 ± 8.7
<65	Increase	5 AF and 2 PC (/22)	24.7 ± 3.5	12.5 ± 6.9

*p < 0.05, **p < 0.01. Comparison of multiple preoperative, intraoperative and postoperative variables for each age group. Patients with any decrease or increase (of 1 point or more) were included. Significant differences are marked in grey.

**Figure 3:**
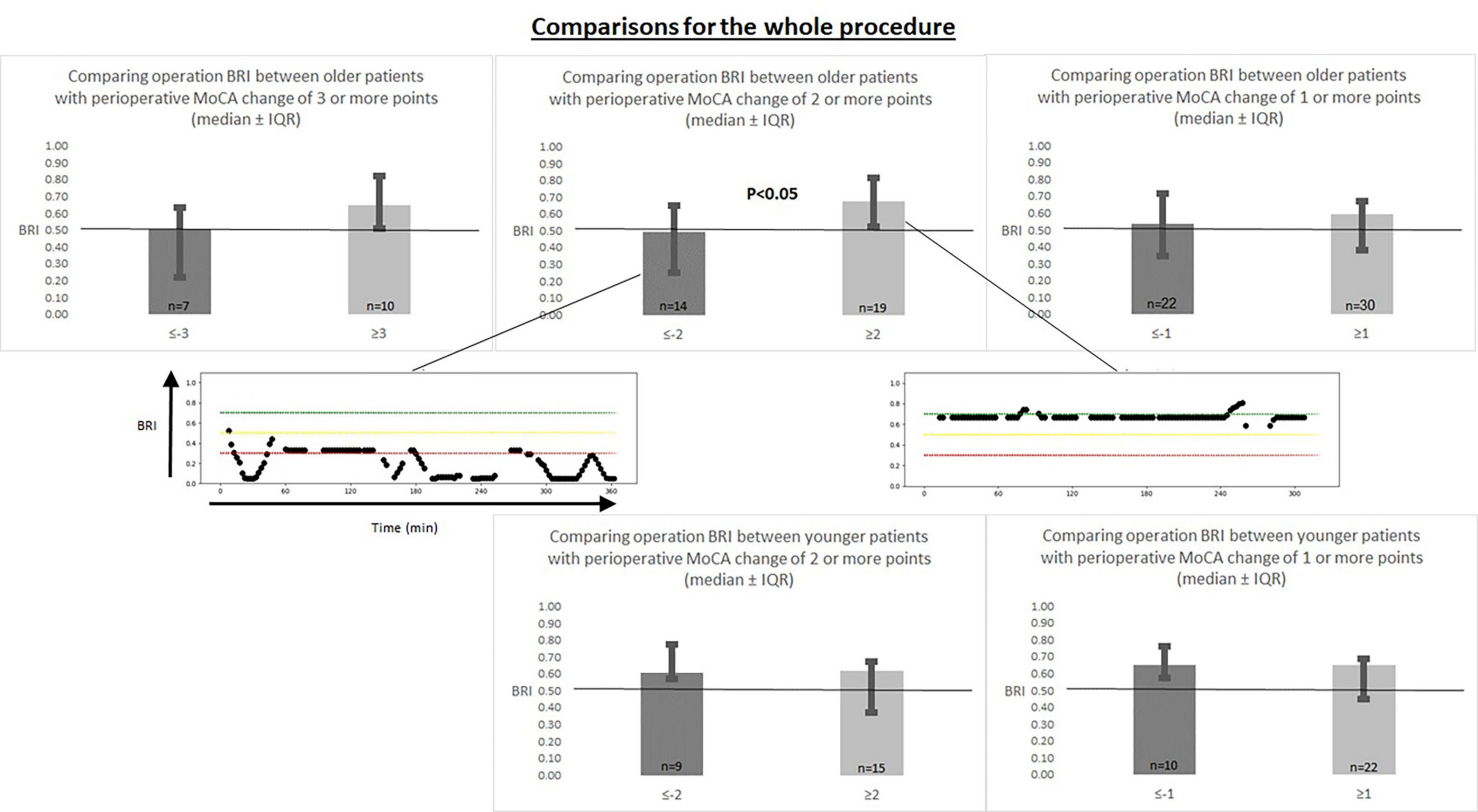
Comparison of the BRI values throughout the procedure by perioperative MoCA change. Each columns graph presents the medians and interquartile ranges of the BRI values for patients with a MoCA decrease and a MoCA increase of at least the specified threshold—the left graph presents the comparison of a threshold of at least 3 points in perioperative MoCA change, the middle graph presents the comparison of a threshold of at least 2 points in perioperative MoCA change, and the right graph presents the comparison of a threshold of any perioperative MoCA change. The upper row presents the comparisons of the older group, and the lower row presents the comparisons of the younger group. Note that in the younger group, the 3-point change comparison is not presented because there were only four patients with at least 3 points perioperative MoCA decrease. A statistically significant difference was found, as shown, only in the older group with at least 2 points perioperative MoCA change. The insets show the BRI values of representative older patients with at least 2 points perioperative MoCA decrease and at least 2 points perioperative MoCA increase.

Figure 3 shows an association between the BRI value throughout the surgery and a perioperative MoCA change of at least 2 points. Older patients who showed a perioperative MoCA decrease of 2 or more points had a significantly lower BRI than patients with a perioperative MoCA increase of 2 or more points (Mann–Whitney U test, z ≈ −2.00, *p* < 0.05). The tendency for this association may also be observed for a change of at least 3 points, but the sample sizes may have been too small to reach statistical significance. The difference appeared to blur when a threshold change of 1 point was considered.

Notably, there was no such significant difference for the younger patients. Among the younger patients, only four experienced a decrease of at least 3 points; therefore, a comparison of changes of at least 3 points was not pursued for this age group.

**Figure 4:**
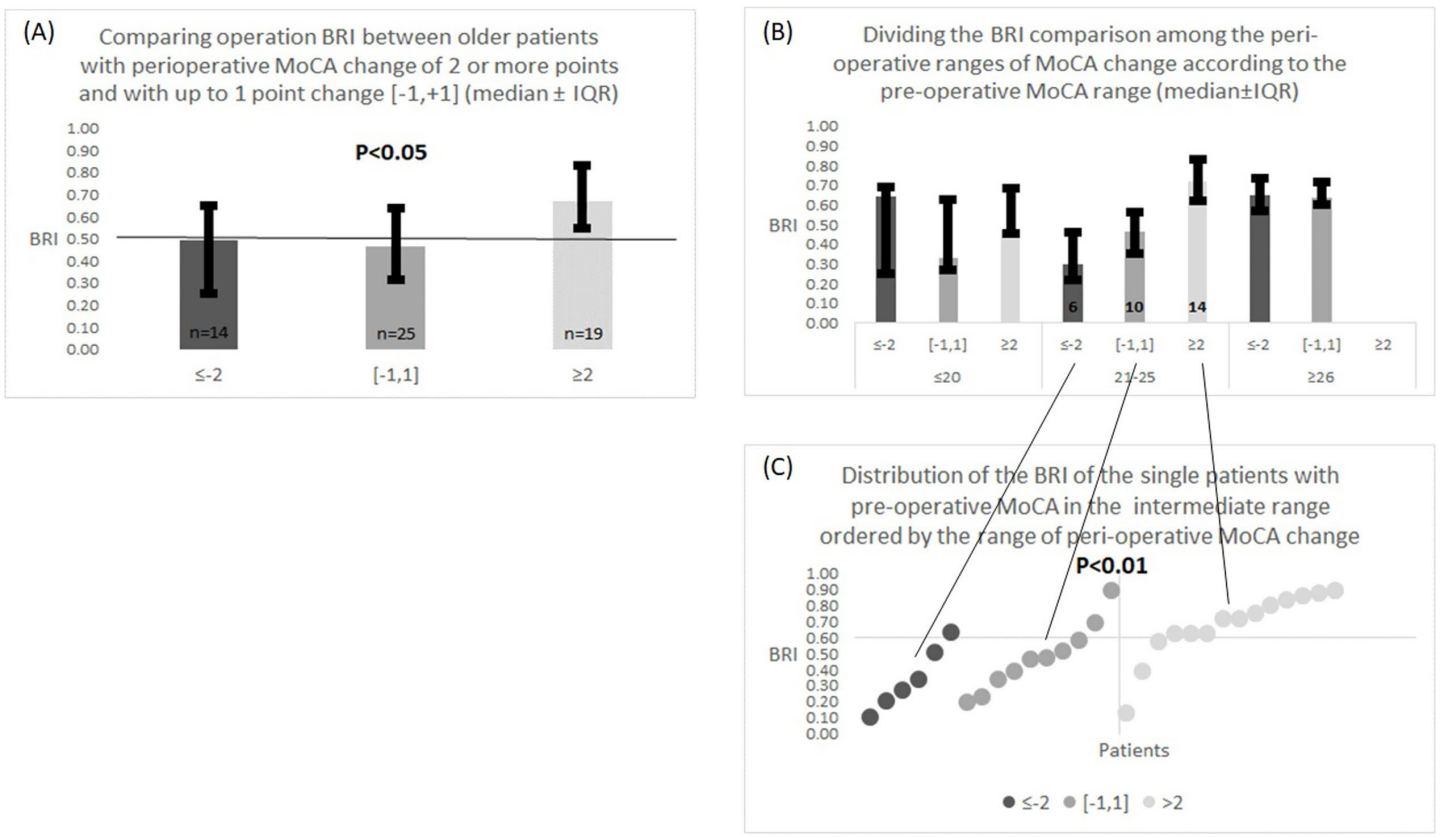
Comparing three ranges of perioperative MoCA change (for three preoperative score ranges). Expansion of the middle upper graph of Figure 3: **(A)** Comparison of BRI medians and interquartile ranges of perioperative MoCA change among the older patients with at least a 2-point decrease, with change between 1 point decrease and 1 point increase, and with at least 2 points increase. The difference was statistically significant. **(B)** Division of the BRI comparison according to preoperative MoCA score range: lower (≤20), intermediate (21–25), and higher (≥26). In the lower and higher ranges, the number of patients in some subgroups of perioperative change seemed too small (below 5) for statistical inference. In the intermediate preoperative MoCA group, the difference in the BRI was statistically significant. **(C)** Distribution of the single patient BRI values in the intermediate preoperative MoCA group with distinction among the perioperative MoCA score ranges of ≤ − 2, [−1,1], and ≥2 (the patients within each sub-group are ordered by BRI value). Three out of 16 patients with perioperative MoCA change in the range of ≤ − 2 and [−1,1] had a BRI value greater than 0.6, and 10 out of 13 patients with perioperative MoCA change in the ≥2 range had a BRI greater than 0.6. This difference was statistically significant.

Following the findings described above of a significant difference in the elderly group between patients with a perioperative MoCA decrease of 2 or more points and patients with a perioperative MoCA increase of 2 or more points, we added the intermediate group of patients with perioperative MoCA change between a decrease of 1 point and an increase of 1 point, including the nine patients without change in their perioperative MoCA scores (Figure 4A). The difference among the three groups was found statistically significant (Kruskal–Wallis test, H ≈ 6.12, *p* < 0.05). Pairwise comparison among the groups did not reach statistical significance after a Bonferroni correction for multiple comparisons.

It is likely that perioperative cognitive change is multifactorial, involving preoperative, intraoperative, and postoperative factors. Even if, as presented in Table 1, we did not find direct associations between other factors and perioperative cognitive change, the combination of factors might be intricate and may not be revealed through single-factor association analyses.

The sample size of this study might be too modest for a thorough multifactorial evaluation, but following previous studies (Baron Shahaf and Shahaf, 2024a), which showed the impact of preoperative MoCA ranges on perioperative cognitive change, we analyzed the potential interaction between the preoperative MoCA range and the intraoperative BRI. We divided the preoperative MoCA score into three ranges: lower (≤20), which may indicate preoperative dementia or at least MCI; higher (≥26), which may indicate normal preoperative cognition; and intermediate (between 21 and 25), which might be indecisive (Baron Shahaf and Shahaf, 2024b). Figure 4B shows the comparison among the three groups of perioperative MoCA change (decrease of 2 or more points, intermediate [−1,1], and increase of 2 or more points) for each preoperative MoCA range. The division of the lower and higher preoperative ranges yielded small numbers of patients in the various perioperative change groups (less than 5), but the intermediate range involved a larger number of patients, and the differences between the groups of perioperative MoCA change in this range were significant (Kruskal–Wallis test, H ≈ 8.16, *p* < 0.05). After a Bonferroni correction, the group with a decrease of 2 or more points differed significantly from the group with an increase of 2 or more points.

As a first assessment of the distribution of the intraoperative BRI for patients in this intermediate preoperative MoCA range across the different groups of perioperative dynamics in the MoCA score, we presented the BRI values of the single patients in these groups (Figure 4C). Thirteen out of 16 patients with a perioperative MoCA decrease of 2 or more points, or from the intermediate MoCA change [−1,1] group, had an intraoperative BRI of less than 0.6 (81%). Only 3 out of 13 patients with a perioperative MoCA increase of 2 or more points also had an intraoperative BRI of less than 0.6 (23%). This difference was statistically significant (Chi-square test, *p* < 0.01).

**Figure 5:**
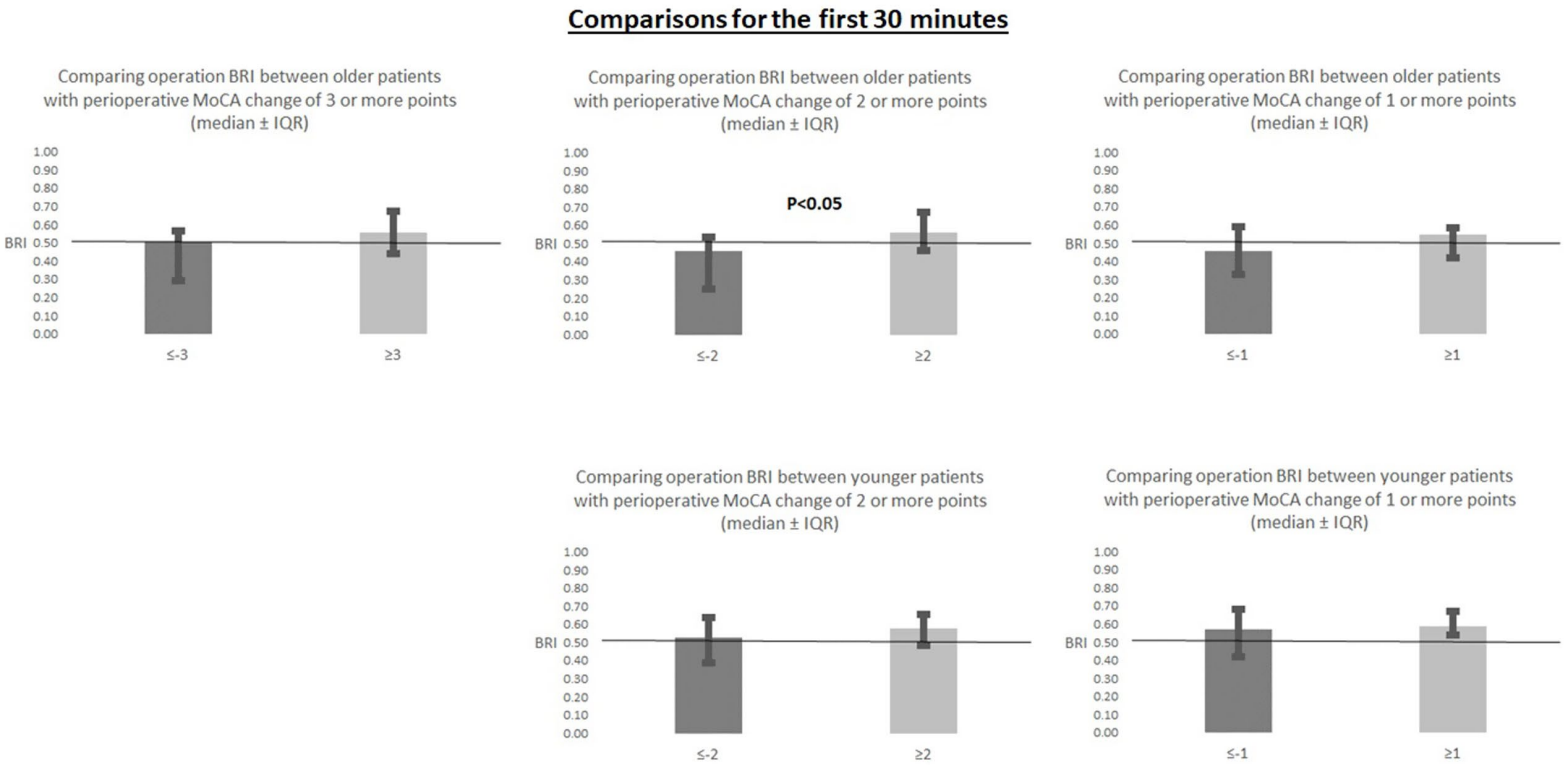
Comparison of the BRI values of the first 30 min by perioperative MoCA change. Each graph presents the medians and interquartile ranges of the BRI values for patients with a MoCA decrease and a MoCA increase of at least the specified threshold—the left graph presents the comparison of a threshold of at least 3 points in perioperative MoCA change, the middle graph presents the comparison of a threshold of at least 2 points in perioperative MoCA change, and the right graph presents the comparison of a threshold of any perioperative MoCA change. The upper row presents the comparisons of the older group, and the lower row presents the comparisons of the younger group. A statistically significant difference was reached, as shown, only in the older group with at least 2 points perioperative MoCA change.

Figure 5 shows that the significant association between the BRI value and a perioperative MoCA change of at least 2 points in the older group was already evident at an early phase of the surgery (Mann–Whitney U test, z ≈ −1.96, *p* < 0.05). The graphs compare the BRI values of the first 30 min of surgery for each threshold change and age group.

## Discussion

The major finding of this study was a possible association between the BRI and a perioperative MoCA change in older patients, but no similar tendency was observed in younger patients. The BRI was found to differ between patients with a MoCA increase of at least 2 pointsand patients with a MoCA decrease of at least 2 points, on the one hand, and potentially also between patients with intermediate perioperative MoCA change between 1 point increase and 1 point decrease, on the other hand. As the same MoCA assessment was repeated within a few days, a performance improvement, e.g., due to improved scores on the memory tasks, could be expected (Duff et al., 2012; Baron Shahaf and Shahaf, 2024b). Lack of such improvement may indicate cognitive compromise.

The association in the older group was evident among the (large) subgroup of patients who had a preoperative MoCA score in the intermediate range (between 21 and 25). In patients with preoperative MoCA scores in the higher range (≥26), a ceiling effect can prevent a perioperative increase in MoCA score by at least 2 points. Conversely, patients with preoperative MoCA scores in the lower range (≤20) tend to undergo perioperative decrease (or at least lack of increase) regardless of their intraoperative BRI. In a previous study (Baron Shahaf and Shahaf, 2024b), we suggested that for patients in this subgroup who demonstrate a perioperative increase, the preoperative score may be an underestimation of their true ability, as they may have focused attention elsewhere and not on the task requirements. Thus, their MoCA score may improve in the postoperative evaluation, but this may be due to the possibility that the preoperative score underestimated their true abilities.

One possible explanation for the lack of similar findings in younger patients may have been a difference in underlying mechanisms that could lead to perioperative MoCA changes. The tendency of a lasting MoCA decline at 6 weeks only in the elderly patients may support such an explanation of distinct mechanisms.

However, the observation that the ratio of patients with a MoCA decrease of 2 points or more is similar in the two age groups might be puzzling. It could be that the impact of a certain underlying cause on perioperative cognitive change increases with age, but if the ratio remains similar, it suggests that the impact of another underlying cause decreases with age. Instead, the similar ratio of MoCA decrease in the two age groups may support a single underlying mechanism.

The BRI was suggested in the introduction to be a potential marker of brain dysfunction during the surgery. It was not suggested as a direct marker of the mechanism that causes cognitive decrease. It might be that the older brain is more sensitive to the underlying mechanism, and its effect on the brain is swifter. However, in the younger brain, the same mechanism might take effect only later and potentially often only postoperatively.

The tendency, noted only for a partially lasting effect in the older group, may lead us to believe that not only the older brain is more sensitive to a swifter effect but also, in a significant ratio of cases, the effect on the older brain might not be transient. Therefore, even the same underlying mechanism would have a different long-term age-dependent effect. However, the similarity in the ratio of decreases of two or more points between the age groups would still remain a puzzle. If the older brain is more susceptible to significant long-term effects, how is it that the ratio of older patients with 2 or more points is not clearly larger?

It is possible that the impact of the specific mechanism that leads to a 2 or more points of MoCA decrease is similar both in the younger and in the older groups, even if it takes longer to impact the younger brain, and its effects may be transient in both cases. However, another long-term mechanism takes effect thereafter in the older group. We have recently suggested that (cognitive) task-related anxiety might lead to a vicious circle of loss of confidence in the older adults following the rather dramatic experience of undergoing surgery (Baron Shahaf and Shahaf, 2024b). Similar to pseudodementia, anxiety may lead to reduced cognitive performance, which may lead to greater anxiety and vice versa. However, this condition of cognitive decline due to anxiety might be treatable, rather effectively, with several focused cognitive rehabilitation sessions (Gvion and Shahaf, 2023), and if this hypothesis is correct, much of the longer-term impact of the decline might be reversible.

Going back to the plausibly transient mechanism for perioperative MoCA change, the BRI might be associated with it as a surrogate marker of intraoperative brain dysfunction in the older group. The significant decrease in the BRI already after the induction of anesthesia seems to indicate that the underlying mechanism, whatever it turns out to be, is more closely related to the anesthesia and less to the surgery. It would require an interventional study to assess whether a decrease in the BRI could be reversed by tuning systemic and anesthetic parameters to prevent delayed neurocognitive recovery in the elderly. It would also be of value to continue the monitoring in the postoperative period in both age groups.

What can the BRI measure? In a previous study, we have suggested that the potential transient cognitive decline in the perioperative period might be due to an attention dysfunction. Delirium is often considered an attention dysfunction (Baron Shahaf and Shahaf, 2024b), and we have suggested (Baron Shahaf and Shahaf, 2024b) that this may also be true for perioperative cognitive decline in general. It seems that under anesthesia, markers of the longer process of sustained attention would be reduced, whereas markers for the shorter process of selective attention may prevail (Baron Shahaf et al., 2020; Baron Shahaf and Shahaf, 2024a). As presented in the introduction section, the BRI was derived from the CEI, a marker for sustained attention (Baron Shahaf et al., 2022), in order to capture such a potentially shorter process. While this hypothesis requires further designated study, it may be that the BRI could be a marker for selective attention, and its decrease, also during surgery, may indicate a risk for attention dysfunction and cognitive decline.

The current study has been only exploratory in nature, with a humble sample size and with the use of only a screening tool for the assessment of perioperative cognitive changes early after the surgery. The validity of the conclusions suggested in the discussion above is heavily dependent on the replication of these findings in larger studies and multiple types of surgery, anesthesia protocols, and thorough cognitive evaluation.

We are happy to provide interested researchers with the BRI marker and our other markers for their studies.

## Data Availability

The original contributions presented in the study are included in the article/supplementary material; further inquiries can be directed to the corresponding author.

## In memoriam

The authors dedicate this manuscript to the memory of Dr. Avishai Ziser, former Head of Cardiothoracic Anesthesia at Rambam Health Care Campus. A mentor, colleague, and a cherished friend. Dr. Avishai Ziser made a significant contribution to the research work and passed away on September 2024. His loss is profoundly felt.
